# *Trans*-[Pt(amine)Cl_2_(PPh_3_)] Complexes Target Mitochondria and Endoplasmic Reticulum in Gastric Cancer Cells

**DOI:** 10.3390/ijms25147739

**Published:** 2024-07-15

**Authors:** Jorge Melones-Herrero, Patricia Delgado-Aliseda, Sofía Figueiras, Javier Velázquez-Gutiérrez, Adoración Gomez Quiroga, Carmela Calés, Isabel Sánchez-Pérez

**Affiliations:** 1 Department of Biochemistry, School of Medicine, Autonomous University of Madrid (UAM), 28029 Madrid, Spain; jmherrero@iib.uam.es (J.M.-H.); pdelgado@iib.uam.es (P.D.-A.); sfigueiras@iib.uam.es (S.F.); javiervelazg@gmail.com (J.V.-G.); ccales@iib.uam.es (C.C.); 2 Instituto de Investigaciones Biomédicas “Sols-Morreale” (IIBM), CSIC-UAM, 28029 Madrid, Spain; 3 Biomarkers and Personalized Approach to Cancer (BioPAC) Group, Area 3 Cancer-Instituto Ramón y Cajal de Investigación Sanitaria (IRYCIS), 28034 Madrid, Spain; 4 Department of Inorganic Chemistry, School of Sciences, Autonomous University of Madrid (UAM), 28049 Madrid, Spain; adoracion.gomez@uam.es; 5 Institute for Advance Research in Chemistry, Autonomous University of Madrid (UAM), 28049 Madrid, Spain; 6 Centro de Investigación Biomédica en Red, Área Rare Diseases, CIBERER-ISCIII, 28029 Madrid, Spain; 7 Unidad Asociada de Biomedicina, UCLM-CSIC, 28029 Madrid, Spain

**Keywords:** platinum complexes, phosphine, gastric cancer, apoptosis, mitochondria, ER stress

## Abstract

Gastric cancer prognosis is still notably poor despite efforts made to improve diagnosis and treatment of the disease. Chemotherapy based on platinum agents is generally used, regardless of the fact that drug toxicity leads to limited clinical efficacy. In order to overcome these problems, our group has been working on the synthesis and study of trans platinum (II) complexes. Here, we explore the potential use of two phosphine-based agents with the general formula *trans*-[Pt(amine)Cl_2_(PPh_3_)], called P1 and P2 (with dimethylamine or isopropylamine, respectively). A cytotoxicity analysis showed that P1 and especially P2 decrease cell viability. Specifically, P2 exhibits higher activity than cisplatin in gastric cancer cells while its toxicity in healthy cells is slightly lower. Both complexes generate Reactive Oxygen Species, produce DNA damage and mitochondrial membrane depolarization, and finally lead to induced apoptosis. Thus, an intrinsic apoptotic pathway emerges as the main type of cell death through the activation of BAX/BAK and BIM and the degradation of MCL1. Additionally, we demonstrate here that P2 produces endoplasmic reticulum stress and activates the Unfolded Protein Response, which also relates to the impairment observed in autophagy markers such as p62 and LC3. Although further studies in other biological models are needed, these results report the biomolecular mechanism of action of these Pt(II)-phosphine prototypes, thus highlighting their potential as novel and effective therapies.

## 1. Introduction

Gastric (or stomach) cancer (GC) is the sixth most frequently diagnosed and the third leading cause of cancer deaths worldwide. In 2020, over a million cases of GC were diagnosed, and the survival rate was notably low, resulting in 768,000 deaths [1,2]. Several factors could contribute to the development of this devastating disease, such as *Helicobacter pylori* infection, smoking, or even obesity [3]. In general, the treatment choice of GC patients is multimodal and involves pre- and postoperative chemotherapy. This strategy has demonstrated better outcomes compared to surgery alone [4]. The perioperative FLOT (5-Fluorouracil, Oxaliplatin, and Docetaxel) regimen is the standard treatment in Europe for locally advanced GC, leading to better results than other approaches in terms of overall survival [5]. However, despite its apparent success, this result may be misleading, as less than half of these patients were able to complete all eight cycles of the perioperative treatment due to toxicity issues [6]. Moreover, resistance is another obstacle associated with classic chemotherapy, provoking clinical failures via tumor relapses. In light of these considerations, the discovery of new drugs that potentially avoid some of these clinical barriers is an obvious public healthcare priority.

One of the possibilities lies in changing the metal center on the treatment molecule. There are numerous examples of new metal-based agents based in gold [7,8], palladium [9,10], copper [11,12], ruthenium [13,14,15], and iridium [16,17], among others. Even though none of these reported better outcomes in GC, platinum(II)-based drugs or their combinations are still leading to good results in research and therefore may have therapeutic potential [18,19,20,21,22]. In fact, nowadays, the most effective clinical platinum drugs are still cisplatin-like derivatives with some structural differences to modulate their reactivity. The *trans* Pt(II) compounds were discarded at the early stages of development due to the lack of therapeutic activity of transplatin. However, it was later reported that in some cases, bulkier ligands binding around the Pt(II) center increased cytotoxicity. Our group reported some of these examples, highlighting the *trans*-[PtI_2_(isopropylamine)_2_] called I5, which leads to great antitumor efficacy in mouse models, avoiding systemic toxicity [23].

The lipophilicity of the ligands coordinated to the Pt atom is a key factor that could favor the intracellular accumulation of the drug. Thus, the coordination of Pt(II) to a lipophilic and bulky ligand leads to a more robust structure, which improves stability, minimizing deactivation processes that could potentially limit the efficacy of the treatment [21] We have reported a series of phosphine-based agents, containing triphenylphosphine ligand (-PPh_3_) and a bulky amine in *trans* configuration, with a general formula *trans*-[Pt(amine)Cl_2_(PPh_3_)] [24]. These compounds exhibited cytotoxic and pro-apoptotic activity (even higher than cisplatin (CDDP)) also against CDDP-resistant cancer cells [24]. We also demonstrated the stability of these complexes at low concentrations in aqueous solutions (1% dimethyl sulfoxide (DMSO)) and using both experimental and computational protocols that allowed us to accurately characterize these complexes in solution.

As triphenylphosphine has been reported to be a mitochondrial targeting group and agents with this ligand are able to modulate mitochondrial metabolism [25,26], we wondered whether the cytotoxicity of our phosphine-based platinum complexes could be mainly due to mitochondrial dysfunction. There are different advantages in targeting mitochondria: (i) inhibiting cancer cell growth (due to the lack of energy); (ii) inducing apoptosis (via cytochrome C and caspases); (iii) bypassing the resistance associated with DNA repair mechanisms; and (iv) disrupting given metabolic processes [27].

In this paper, we have explored the mechanisms of action of two phosphine complexes: P1 (*trans*-[PtCl_2_(dimethylamine)(PPh_3_)]) and P2 (*trans*-[PtCl_2_(isopropylamine)(PPh_3_)]). We have measured their potential cytotoxicity in various gastro-intestinal tumor cell lines and in the non-tumoral Human Pancreatic Duct Epithelial (HPDE) cell line. We also included in our studies a healthy cell line from a different tissue, AC16 primary cardiomyocytes, to gauge the toxicity or specificity of these compounds in other tissues, specifically the heart, and finally conducted a further characterization analysis on GC cells. We have thoroughly evaluated mitochondrial status and Reactive Oxygen Species (ROS) generation, DNA damage, and endoplasmic reticulum (ER) stress. Altogether, the results we present here show a pivotal role for mitochondrial apoptosis in the mechanism of cell death that these complexes have shown to induce in cellulo.

## 2. Results

### 2.1. Phospine-Based Agents P1 and P2 Decrease Cell Proliferation

**Figure 1:**
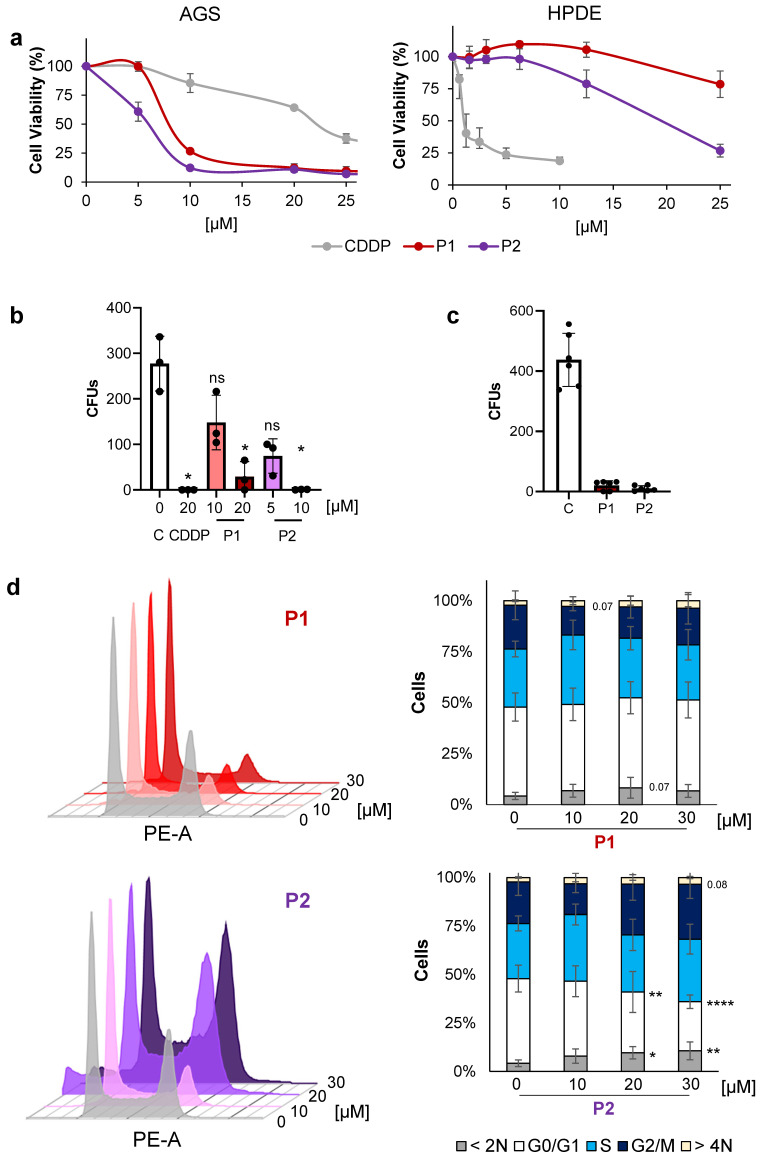
Phosphine agents decrease cell proliferation. (**a**) Cell viability studies with P1 (red), P2 (purple), and CDDP (grey) used as a positive control in GC AGS cells (left side) and in healthy HPDE cells (right side) after 48 h of treatment. The cells were treated with increasing concentrations of either agent (0–25 µM). The percentage of viable cells was quantified by an MTS assay. The data represent the mean values obtained in three experiments performed in quadruplicate. (**b**) A Colony Forming Unit (CFU) assay was used to determine cell clonogenicity. The AGS cells were treated with CDDP (20 µM), P1 (10 and 20 µM), or P2 (5 and 10 µM), and the colonies that formed were stained with crystal violet and quantified 10 days later. (**c**) The AGS cells were pre-treated with CDDP (20 µM), P1 (20 µM), and P2 (10 µM) for 3 h. Then, the cells were counted and seeded (see the Section 4), and the colonies were then stained with crystal violet and quantified 10 days later. In both CFU assays, statistical significance was evaluated by one-way ANOVA with a Dunnett post-test (ns = not significant, * *p* < 0.05) compared to the untreated cells (C: control). N = 3. (**d**) The AGS cells were treated with increasing concentrations of P1 and P2 (10, 20, and 30 µM). Twenty-four hours after the treatments, the cells were fixed and stained with propidium iodide. The graphs show the cell cycle profile after the treatments (left panel) and the percentage of cells in each phase (right panel). Apoptosis was quantified as the percentage of cells with DNA content < 2N. Statistical significance was evaluated by one-way ANOVA followed by a Dunnett post-test (* *p* < 0.05, ** *p* < 0.01, **** *p* < 0.0001) compared to the untreated cells (C: control). N = 3.

We first assessed the cytotoxic effects of P1 and P2 on both tumoral (AGS, MKN45, and PANC1) and non-tumoral cell lines (AC16 and HPDE). To that end, cells were exposed to increasing concentrations (0–25 μM) of P1, P2, or CDDP for 48 h. The obtained IC_50_ values (Table 1) indicated that both complexes decreased cell viability in a dose-dependent manner (Figure 1 and Figure S1a), with P1 and P2 demonstrating greater activity in AGS tumoral cells compared to CDDP, a standard clinical treatment. Regarding selective indexes between gastrointestinal tumor cells and HPDE, it is noteworthy that non-tumoral cells exhibited lower sensitivity to the phosphine agents than to CDDP (Table 1). In addition, P2 revealed higher cardiotoxicity than P1, but in both cases, lower than CDDP (see the selective indexes in Table S1).

To further study the effect of our candidate agents on cell proliferation, we evaluated the impact of P1 and P2 on clonogenicity. The Colony Forming Unit (CFU) analysis revealed that both complexes inhibit proliferation at the IC_50_ concentration or higher (Figure 1b and Figure S1b). We also observed that 3 h of treatment is enough to prevent colony formation, and this effect is still evident after removing the agent and maintaining the growth of cells for 10 days in a drug-free medium. This effect is similar to that of cells subjected to a maintained treatment over the course of 10 days (Figure 1c and Figure S1c). To evaluate if the effect on proliferation correlated with cell mobility processes, net, we studied cell migration after our treatment options but failed to detect any significant effect (Figure S2).

Based on the results and before continuing with studies on the mechanism of action, we evaluated whether the cells had taken up the drugs. To do this, we quantified the concentration of platinum inside AGS cells treated with P1 and P2 (10 µM) for 3 h. We observed that after 3 h treatment, although platinum is found in both the nucleus and cytoplasm, both complexes preferentially accumulate in the cytoplasmatic fraction, with higher uptake observed in P2 samples (Table 2).

We then analyzed the cell cycle profile after 24 h of treatment with P1 and P2 in AGS cells. We observed that both complexes, notably P2, induced apoptosis. Additionally, P2 slightly increased the percentage of cells in the S and G2/M phases more efficiently than P1 (Figure 1d, Table S2). These results indicate that none of the complexes affects cell mobility processes. However, both do interfere with GC cell viability and with proliferation.

### 2.2. P1 and P2 Generate ROS and Produce DNA Damage

**Figure 2:**
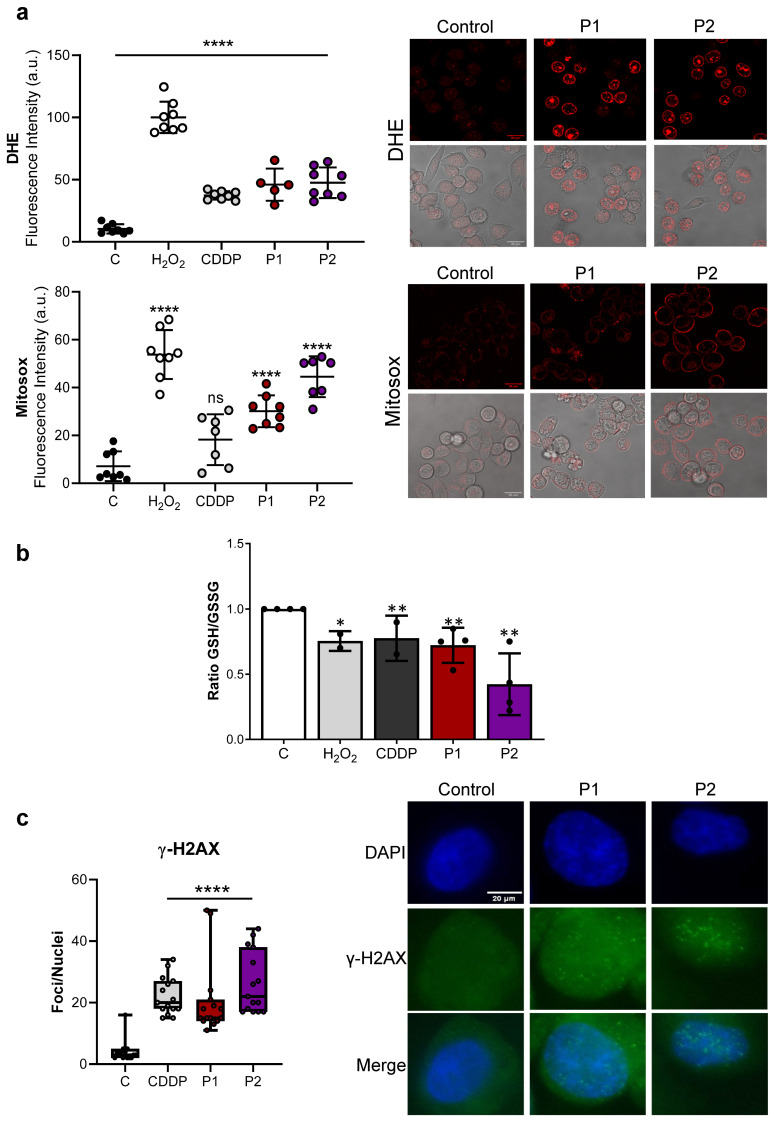
P1 and P2 generate ROS and cause DNA damage. (**a**) The detection of ROS in AGS cells after treatment with H_2_O_2_ (positive control, 200 µM), CDDP (10 µM), P1 (20 µM), and P2 (10 µM) by confocal microscopy using DHE and MitoSox as O_2_^•−^ Red fluorescence indicator in cytosol and mitochondria, respectively. The cells were treated with the compounds for 1 h followed by 30 min of incubation with the probes. Representative images of each condition were taken (H_2_O_2_ and CDDP are included in Figure S3b) in fluorescence (upper panel) and brightfield (lower panel) conditions. The scale bar represents 20 μm. Fluorescence intensity (per cell) was quantified and depicted in the graph. Statistical significance was evaluated by one-way ANOVA followed by a Dunnett post-test (ns = not significant, **** *p* < 0.0001) compared to the untreated cells (C: control). N = 3. (**b**) The ratio of GSH/GSSG determined with a commercial colorimetric kit (see the Section 4). The AGS cells were treated with the IC_50_ concentration of the compounds and were collected after 24 h to perform the assay. N = 3. Statistical significance was evaluated with Student’s 2-tailed *t*-test (* *p* < 0.05, ** *p* < 0.01) compared to the untreated cells (control). (**c**) The AGS cells were treated with CDDP (20 µM), P1 (20 µM), and P2 (10 µM) for 3 h. γ-H_2_AX foci (green fluorescence) were detected by immunofluorescence using DAPI to stain nuclear DNA (blue fluorescence). Representative images of each condition were taken (Scale bar: 20 µm). The graph represents the number of foci per nuclei for each condition. Statistical significance was evaluated by one-way ANOVA followed by a Dunnett post-test, **** *p* < 0.0001) compared to the untreated cells (C: control). N = 3.

Platinum complexes increase cellular ROS [23]. Therefore, we aimed to investigate whether the phosphine-based agents generated ROS in vitro. We first quantified the levels of superoxide anions (O_2_^•−^) in the cytosol of living cells (using Dihydroethidium (DHE) in AGS and MKN45 cells) and in mitochondria (using Mitosox in AGS cells) after 1 h of treatment with P1 (20 µM) and P2 (10 µM) but also with CDDP (20 µM) and H_2_O_2_ (200 µM), which were used as positive controls. The signal obtained from our fluorescence indicator rapidly increased with both complexes, being slightly higher with P2, especially regarding mitochondrial ROS species (Figure 2a and Figure S3a,b). Subsequently, we investigated the GSH/GSSG system to further assess the redox status induced by P1 and P2. The higher presence of oxidized glutathione (GSSG) serves as an indicator of the presence of oxidant species that react with reduced glutathione (GSH). Thus, a lower GSH/GSSG ratio correlates with a higher ROS concentration in cells. We observed a decrease in this ratio after a 24 h treatment with both of our agents (Figure 2b).

To evaluate the influence of ROS on genotoxic stress, we analyzed the DNA double-stranded breaks in the AGS cells. The quantification of H2AX^Ser139^ positive nuclei (Figure 2c and Figure S3c) showed that the number of foci per nucleus significantly increased 3 h after treatment (like CDDP) on a range of 20 to 40 foci per nuclei. Next, we evaluated the phosphorylation of p53 or CHK1 as markers of DNA damage response. While CDDP increased the activation of both, P1 and P2 only slightly activated these kinases (Figure S4a,b).

These results suggest that, in GC cells, P1 and P2 are more effective than CDDP in generating ROS mainly in the mitochondria, which potentially leads to oxidative DNA damage.

### 2.3. P1 and P2 Target Mitochondria and Induce Apoptosis

**Figure 3:**
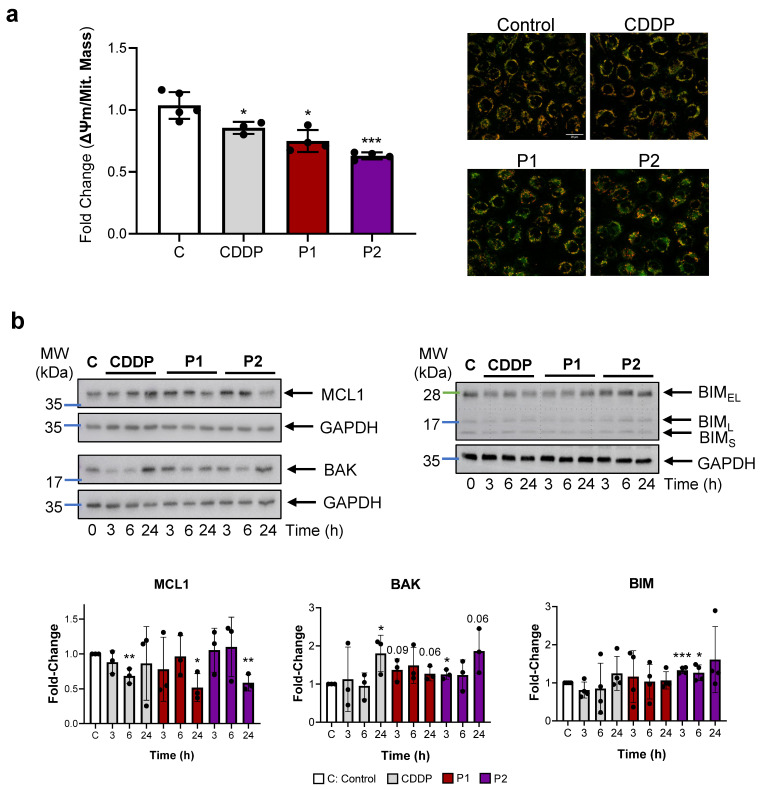
P1 and P2 target mitochondria and induce intrinsic apoptosis. (**a**) Phosphine agents produce mitochondrial dysfunction in AGS GC cells. Mean fold-change ± SD of the ratio of the ΔΨm probe CMX-ROS (Red) versus the Mitochondrial Mass probe MitoGreen (Green) as a measurement to evaluate mitochondrial functionality in AGS cells treated for 2 h with CDDP (10 µM), P1 (20 µM), and P2 (10 µM). Representative images for each condition were taken (CDDP was used as a positive control). The scale bar represents 20 μm. Fluorescence intensity (per cell) was quantified and depicted in the graph. * *p* < 0.05, *** *p* < 0.001, as determined by one-way ANOVA followed by a Dunnett post-test, compared to the control, set as 1.0. N = 3. (**b**) Western blot analysis of mitochondrial apoptosis: MCL1 and BAK (left panel), and BIM (right panel). Representative Western blots in AGS cells after treatment with IC_50_ concentration of CDDP, P1, or P2 at different times (3, 6, and 24 h). GAPDH was used as an endogenous loading control. The graphs show the mean ± SD densitometric analyses of each protein normalized with GAPDH from three independent experiments by using ImageJ (area under the peak method), control cells (C, white bars), CDDP (grey), P1 (red), and P2 (purple). Statistical significance was evaluated with Student’s 2-tailed *t*-test (* *p* < 0.05, ** *p* < 0.01, *** *p* < 0.001) compared to the untreated cells (C: control), set as 1.0.

Excessive levels of ROS in the mitochondria affect their membrane potential (ΔΨm). We studied the status of the mitochondria by measuring mitochondrial mass and ΔΨm in AGS cells exposed to CDDP, P1, and P2 (IC_50_ concentration) for 2 h. Mitochondrial mass and ΔΨm were measured by using MitoTracker™ Green and MitoTracker™ Red CMXRos, respectively, and used to calculate the ΔΨm/Mit. Mass ratio, which allows the membrane potential respective to the total mitochondrial mass to be normalized (Figure 3a). Our results show that CDDP, P1, and P2 decrease the ΔΨm/Mit. The mass ratio indicates the presence of dysfunctional mitochondria.

Next, we studied the effect of the permeabilized mitochondria on the intrinsic apoptotic pathway after treatment with these complexes. We evaluated the expression of different BCL2 family member proteins: (i) pro-apoptotic—BAX, BAK, and BIM; (ii) anti-apoptotic—MCL1. All of the complexes (CDDP, P1, and P2) induced a reduction in MCL1 expression after 24 h. By contrast, CDDP and P2 treatment led to an increased BAK expression and only P2 significantly increased the levels of BIM, while P1 preferentially increased BAX expression (Figure 3b and Figure S4c). Altogether, these results underline mitochondrial apoptosis as a possible scenario of cell death elicited by these agents.

### 2.4. P1 and P2 Produce ER Stress and Block Autophagy

**Figure 4:**
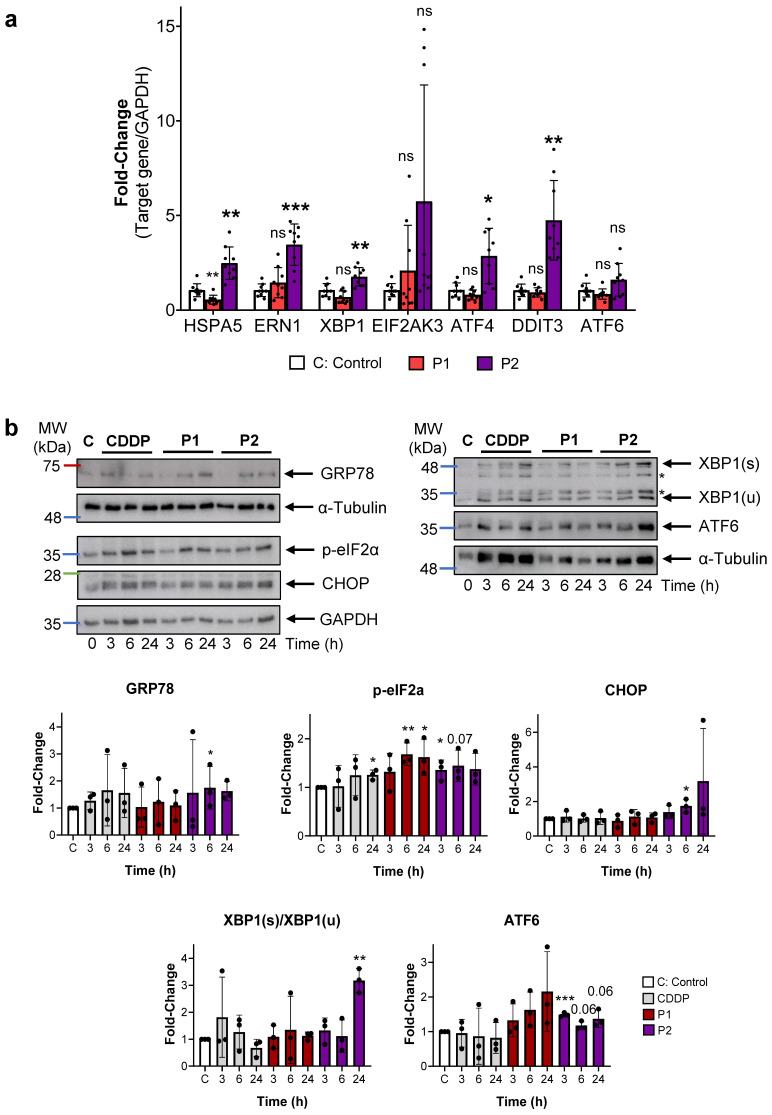
P2 produces ER stress. (**a**) RNA was isolated from the AGS cells stimulated with a 24 h treatment of the complexes. HSPA5, ERN1, XBP1, EIF2AK3, ATF4, DDIT3, and ATF6 were quantified by RT-qPCR. Target gene expression was normalized to GAPDH. All experiments were performed three times per triplicate with IC_50_ concentrations of each compound used in all the assays. Statistical significance was evaluated by Student’s 2-tailed *t*-test (ns: not significant * *p* < 0.05, ** *p* < 0.01, *** *p* < 0.001) compared to untreated cells (C), set as 1.0. (**b**) Western blot analysis of reticulum stress proteins: GRP78, p-eIF2α, CHOP, XBP1 * inespecific bands), and ATF6. GAPDH or α-Tubulin was used as endogenous loading controls. The graphs show the mean ± SD densitometric analyses of each protein normalized with GAPDH from three independent experiments by using ImageJ (area under the peak method), control cells (C, white bars), CDDP (grey), P1 (red), and P2 (purple). Statistical significance was evaluated by Student’s 2-tailed *t*-test (* *p* < 0.05, ** *p* < 0.01, *** *p* < 0.001) compared to the untreated cells (C: control), set as 1.0.

The ER has emerged as a promising target for anticancer metallodrugs with redox activity [28]. ROS induce protein misfolding, thus triggering the Unfolded Protein Response (UPR). Cells treated with CDDP, P1, and P2 were analyzed for a set of genes representative of each related pathway: PERK (EIF2AK3, ATF4, and C/EBP homologous protein (CHOP), encoded by the DDIT3 gene); IRE1α (ERN1 and XBP1); ATF6 (Activating Transcription Factor 6); and the common unfolded protein receptor from all three pathways, 78 kDa glucose-regulated protein (GRP78), encoded by the HSPA5 gene. The RT-PCR analysis revealed a robust induction of IRE1α and PERK-related genes in cells treated with P2, while no significant induction was observed after P1 treatment (Figure 4a). To confirm protein induction, we analyzed by Western blotting GRP78, phosphorylated eukaryotic translation initiation factor 2α (p-eIF2α), CHOP, X-Box Binding Protein 1 (XBP1), and ATF6, all closely associated with ER stress and apoptosis. Our results confirmed that while all treatments induce the phosphorylation of eIF2α, only P2 leads to increased levels of all examined proteins (including the alternatively spliced form of XBP1), which is consistent with our qPCR findings (Figure 4b).

**Figure 5:**
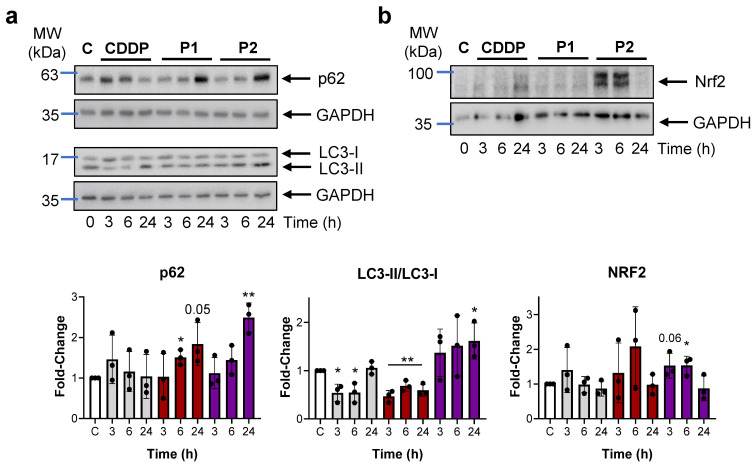
(**a**,**b**) P1 and P2 impair autophagy. Western blot analysis of autophagy: p62 and LC3, and Nrf2. Representative Western blots in AGS cells after the treatment with IC_50_ concentration of CDDP, P1, or P2 at different time points (3, 6, and 24 h). GAPDH was used as an endogenous loading control. The graphs show the mean ± SD densitometric analyses of each protein normalized with GAPDH from three independent experiments by using ImageJ (area under the peak method), control cells (C, white bars), CDDP (grey), P1 (red), and P2 (purple). Statistical significance was evaluated with Student’s 2-tailed *t*-test (* *p* < 0.05, ** *p* < 0.01) compared to the untreated cells (C: control), set as 1.0.

Autophagy and UPR are activated in stressed cells in order to restore their cellular homeostasis. Consequently, we investigated the impact of P1 and P2 treatment on autophagy markers in AGS cells. To this end, we evaluated p62 and microtubule-associated protein 1 light chain 3 alpha (LC3-I and LC3-II) by Western blotting. LC3-I is a cytosolic form that, after activation by Atg7, is transferred to Atg3 and finally modified to LC3-II, the active membrane-bound form, typically located in the autophagosomes [29]. The results from this set of experiments indicate that P2 influenced autophagy by increasing p62 and LC3-II (Figure 5). Finally, due to the known role of p62 in redox signaling after oxidative conditions, we sought to determine Nrf2 (nuclear factor erythroid 2-related factor 2) expression in our cells by Western blotting. Our blots showed that both P1 and P2 increase Nrf2 levels, with this increase being coupled to p62 accumulation [30].

## 3. Discussion

The approval of CDDP as an antitumor drug initiated a long search for new candidates with a potential to overcome the clinical challenges that limit CDDP-based chemotherapy. Carboplatin and oxaliplatin achieved the goal of being approved worldwide [31]. The development of more effective drugs with less side effects is still a healthcare priority due to the high incidence and the elevated death rate associated to cancer diseases.

Phosphine-based platinum(II) complexes have gained attention due to the possibility of targeting mitochondria and the ability to overcome CDDP resistance [24,25,26]. Herein, we start the preclinical stage studies of the prototypes *trans*-[PtCl_2_(dimethylamine)(PPh_3_)] (P1) and *trans*-[PtCl_2_(isopropylamine)(PPh_3_)] (P2) against GC. In our studies, we do find cytotoxic effects on gastrointestinal cancer cells, and we show that, at least in GC cells, this occurs through the intrinsic apoptotic pathway. However, while both P1 and P2 disrupt mitochondrial potential and generate ROS in GC cells, only P2 activates UPR, ER stress, and autophagy. This highlights the fact that their mechanism of action may differ, possibly due to differences in the chemical residues of both complexes.

Our group has previously demonstrated the apoptotic effect of these compounds in ovarian cancer cell lines, either sensitive or resistant to CDDP (CH1 and CH1cisR) [24]. Other studies with phosphorus donor ligands exhibit this capability to overcome CDDP resistance in breast cancer [32,33]. Here, we document the impact of these compounds in GC cells. P2, which contains the bulkiest amine (isopropylamine), demonstrated the highest activity and cell uptake. Although in gastrointestinal healthy cells, both complexes exhibited less cytotoxicity than in cancer cells, other systemic toxicity issues (such us cardiotoxicity observed in AC16 cells) should be explored.

Mitochondria are crucial players in cancer cell survival, as they are the bioenergetic and biosynthetic hubs that coordinate cellular respiration, the tricarboxylic acid cycle, Ca^2+^ signaling, and redox homeostasis. Emerging evidence strongly indicates that resistant tumor cells exhibit high mitochondrial respiration and oxidative phosphorylation status [34]. Therefore, targeting mitochondria represents a promising cancer treatment avenue and chemoresistance overcoming strategy. Due to the presence of a triphenylphosphine ligand, we studied the mitochondrial status in our cell lines, detecting an abnormal function in their mitochondrial network, which is related to depolarization processes. The introduction of a metal complex together with mitochondrial damage, could increase ROS levels [23]. CDDP has been reported as ROS inductor, also linked to its capability to damage DNA. P1 and P2, besides ROS production, produce DNA damage as well, detected by the phosphorylation of H2A at ser139 (usually referred as γ-H2AX). Genotoxicity related to ROS production has been observed with other platinum [35,36,37] and non-platinum complexes containing phosphine-derived ligands, such as Pd(II) [38] or gold(I/III) [39].

The apoptotic effect of different platinum complexes linked to phosphorus donor ligands has been reported over the years [25,32,33,35,36,40]. However, the mechanism of action of P1 and P2 complexes seems to be independent of p53, in agreement with previous results in HCT116, the wild type, and p53 knockout [41]. This is of particular interest because more than 50% of all solid tumors have mutated p53 [42], so chemotherapy based on p53 would fail in these patients, while a p53-independent therapy could be the best treatment avenue for them.

Stress factors such as free radicals or ER stress permeabilize the outer mitochondria membrane typically after the activation of specific proapoptotic members of the BCL-2 family [43,44]. We demonstrate here that P1 and P2 both induce intrinsic apoptosis via BAX/BAK: after oligomerization, they make the mitochondria permeable so that it releases apoptotic factors to the cytosol such as cytochrome c or SMAC/DIABLO, which in turn will activate the caspase cascade. Moreover, we observed a reduction in MCL1 expression that positively correlated with the described apoptotic effect. These results support our previous studies using CDDP [45]. Only P2 induces BIM expression. BIM induction is closely related to apoptosis via ER stress, with reports highlighting the connection between the transcription factor CHOP and the proapoptotic Bcl-2 family member BIM [46,47]. Accordingly, only P2 induces CHOP and, thus, ER stress and apoptosis via BIM.

The accumulation of unfolded proteins that contribute to ER stress could also be responsible for autophagy imbalance [48,49]. In standard conditions, autophagy should target misfolded proteins and damaged organelles to be degraded by the autophagosomes, thus preventing cells from suffering ER and/or oxidative stress [50]: the presence of LC3-II is considered an autophagy marker. Our data show that the ratio LC3-II/LC3-I only increases after treatment with P2. The expected trend for the expression of autophagy-inducing agents is to detect that the increase in LC3-II parallels a reduction in p62 levels, since after interaction with LC3, p62 is degraded by the autophagosome-lysosome system. The ablation of autophagy then results in the accumulation of p62 [51]. While this scenario seems to be the result of CDDP and P1 treatment (with reduced levels of LC3-II and an increase in p62), this is not the case with P2, which in fact resulted in higher levels of both proteins. A possible explanation for this could be related to the role of p62 after oxidative stress conditions in recruiting detoxification enzymes mainly via Nrf2 [50,52] to maintain cellular homeostasis.

**Figure 6:**
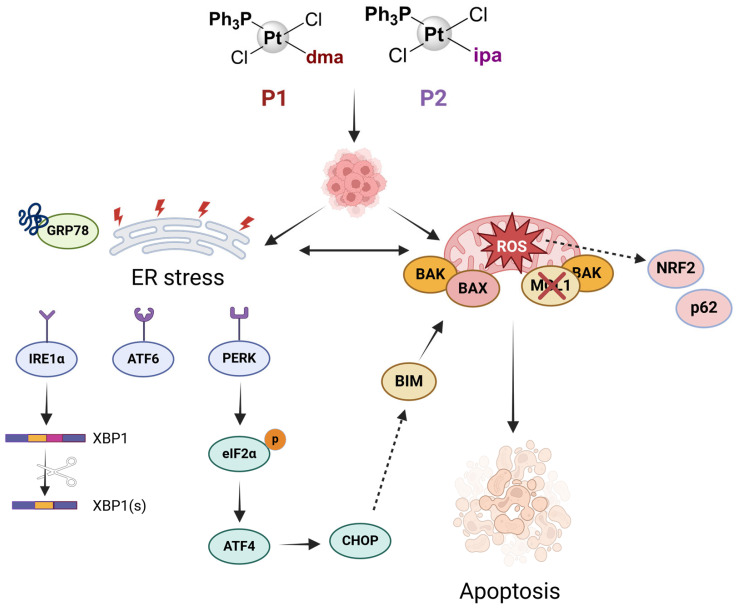
Hypothetical model of P1 and P2 mechanism of action. Created using biorender.com https://www.biorender.com/ (accessed on 28 June 2024).

Finally, we propose a model for the mechanism of action of P1 and P2 in which both compounds induce ROS primarily in the mitochondria. This leads to the loss of mitochondrial membrane potential, culminating in mitochondrial dysfunction. As a consequence, misfolded proteins start to accumulate, and this triggers the UPR response in an attempt to maintain cellular homeostasis. The activation of transcription factors CHOP and XBP1 then promotes the expression of proapoptotic proteins Bim and BAK, ultimately resulting in cell death via apoptosis (Figure 6). Although further studies are needed for a deeper understanding of the full mechanism of action of these complexes, the results we describe here for P1 and P2 encourage us to start thinking about the impact of their potential effects in other biological and pre-clinical models.

## 4. Materials and Methods

### 4.1. Cell Culture

The human gastric adenocarcinoma cell lines MKN45 (poorly differentiated; DSMZ: Deutsche Sammlung von Mikroorganismen und Zellkulturen GmbH, Leibniz-Institut DSMZ, Braunschweig, Germany) and AGS (primary tumor; ATCC/LGC Standards, Spain) were cultured in RPMI 1640 (Sigma San Luis, MO, USA) and HAM’S F12 ((Sigma, San Luis, MO, USA), respectively. The pancreatic cell line PANC1 (ATCC) was cultured in RPMI 1640 (Sigma). Control normal cell lines AC16 (cardiomioblasts, ATCC) and HPDE (Human Pancreatic Duct Epithelial cells, ATCC) were cultured in RPMI ((Sigma, San Luis, MO, USAs). RPMI was supplemented with 10% Fetal Bovine Serum, 2 mM L-Glutamine, Fungizone (2.5 μg/mL), and Gentamicin (0.035 mg/mL). The cells were cultured at 37 °C, 5% CO_2_, and 95% humidity. Mycoplasma contamination tests are frequently run in our laboratory. The experiments were performed between passages two to eight.

### 4.2. Chemicals

CDDP was supplied by Ferrer FARMA(Ferrer Internacional, S.A. Avenida Diagonal 549, 08029 Barcelona (Spain). The compounds used for the assays (P1 and P2) were synthesized following previously reported procedures [24]. In all the experiments, the cells were treated with the metallodrugs following an appropriate sample preparation protocol: CDDP was dissolved in water, and P1 and P2 in DMSO and then immediately diluted with the culture medium to the appropriate concentration, with a final DMSO concentration of 1%. The control cells were also maintained in 1% of DMSO-supplemented cell culture medium.

### 4.3. Cell Viability

Cell viability was assessed using an MTS (Promega Promega Biotech Ibérica SL, Madrid, Spain) staining method. Cells (5 × 10^3^) were seeded per well in 96 multi-well plates. The following day, the cells were treated with selected compounds at different concentrations (0–25 µM). After 48 h, 10 µL/well MTS solution was added to each well, and after incubation in the darkness for 1–4 h (37 °C, 5% CO_2_), absorbance was recorded at 490 nm. IC_50_ values were calculated by using GraphPad Prism (version 8.0). Nonlinear regression was used to fit the data to the log (inhibitor) versus response (variable slope).

### 4.4. Clonogenic Assay

For the clonogenic assay, we used two different approaches. (i) AGS cells were cultured in a 6-well plate at a very low concentration (2 × 10^3^ cells per well), incubated overnight to ensure attachment, and then treated with IC_50_ and a higher concentration (2·IC_50_) of the compounds and incubated for 10 days. (ii) AGS cells were treated with the metallodrugs for 3 h. Then, the cells were cultured in a 6-well plate at a very low concentration (2 × 10^3^ cells per well) and incubated for 10 days. Then, the cells were fixed with 1% glutaraldehyde and stained with 0.1% crystal violet. The number of colonies was quantified directly using the 10× objective Nikon Eclipse TS 100 microscope Nikon Instruments Inc., Melville, NY, USA) in three different areas per well. Representative images were taken in the IIBM image facility (NIKON COOLPIX5700 camera, Nikon Instruments Inc., Melville, NY, USA).

### 4.5. Wound Healing (Cell Migration) Assay

Cell migration was also studied using the wound healing method. A wound healing assay was used to study the effects of P1 (10 µM), P2 (10 µM), and CDDP (10 µM) in two gastro-intestinal cellular model systems: MKN45 (gastric cancer) and PANC1 pancreatic cancer) cells. The cells were cultured to 80% confluence in 24-well plates (7 × 10^5^ cells/0.5 mL). Subsequently, the cell layer was scratched with a sterile 10 µL pipette tip. We removed the medium and added 500 µL of an appropriate concentration of the drugs. The migratory ability of the treated cells was monitored from 0 h to 48 h with a microscope (Observer Z1, Zeiss, Carl Zeiss Microscopy GmbH, Jena, Germany) and compared to the non-treated cells.

### 4.6. Cellular Uptake and Distribution by ICP-MS

To assess the uptake and the intracellular distribution of P1 and P2, AGS cells were seeded in p60 plates (1.5 × 10^6^ cells per plate) and allowed to attach overnight (37 °C, 5% CO_2_). The adherent cells were incubated with the drugs (10 µM) for 3 h, and then washed with PBS and tripsinized. In the suspension, the cells were counted, so that 10^6^ cells were separated, washed twice with cold PBS, and centrifuged (1000 rpm, 5 min) to obtain a pellet. To disrupt the cellular membrane, the pellet was resuspended in a lysis buffer (10 mM Tris, 1.5 mM MgCl_2_, 140 mM NaCl, pH 8.0–8.3) with Nonidet P40 (0.02%) [45]. After 15 min of incubation on ice, the suspension was centrifuged at 1300× *g* for 2 min at 4 °C and the nuclear fraction (pellet) was separated from the cytoplasmatic fraction (supernatant). The Pt content in the two fractions was measured after digestion in an open vase with HNO_3_ (65%) and HCl, evaporated, and resuspended in ultrapure water to obtain a 2.0% (*v*/*v*) nitric acid solution using an ICP-MS aurora Elite BRUKER with ^187^Rhenium used as internal standard. The protocol was performed at the Geological Techniques Unit of Assistance Centers for Research at the UCM, which fulfill the Quality Management System IQNet—UNE-EN-ISO 9001:2015 (Spain) [53].

### 4.7. Cell Cycle Profile Analysis

We analyzed the effect of 24 h of treatment with various concentrations of our complexes P1 and P2 (10, 20, and 30 µM) on cell cycle. The cells were fixed with 70% cold EtOH and stored at 4 °C overnight. The cells were then incubated with Propidium Iodide (50 µg/mL) and RNAse (10 µg/mL) for 30 min, and their DNA content was evaluated using a FACS CANTOII Flow Cytometer (Becton Dickinson Rowa, San Agustin de Guadalix, Madrid, Spain). The results were analyzed using FACS Diva Software FACS Diva v9.0 and FlowJo v10. Apoptosis was quantified as the percentage of cells with DNA content < 2 N (Sub G1). The gating strategy used in all the Flow Cytometry experiments is specified in Figure S5.

### 4.8. ROS Detection by Confocal Microscopy

GC cells were seeded at a density of 3 × 10^5^ cells in 15 µm 8-well IBIDI plates and incubated overnight. The cells were then treated with compounds at their IC_50_ concentrations and incubated for 1 h. Subsequently, the cells were stained with 10 μM of DHE (cytosolic O_2_^•−^ probe, Sigma-Aldrich, Burlington, UK) or with 5 μM of Mitosox (mitochondrial O_2_^•−^ probe, Sigma-Aldrich) for 15–30 min and then visualized on a confocal microscope LSM710, Zeiss, objective plan-apochromatic 63× (IIBM-CSIC-UAM), in fluorescence and brightfield conditions. The image analysis was performed using the software program ImageJ 1.54f.

### 4.9. Ratio GSH/GSSG

The GSH and GSSG levels were determined in the AGS cells after 24 h of treatment with the compounds (CDDP 20 µM, P1 20 µM, P2 10 µM, and using H_2_O_2_ 200 µM as a positive control) with the Glutathione Colorimetric Detection Kit (ThermoFisher, Waltham, MA, USA) following the manufacturer’s instructions. Thirty minutes after adding the Colorimetric Detection Reagent and the Reaction Mixture, the absorbance was read at 405 nm. A standard curve was generated with each assay to interpolate the sample results. To measure GSSG, standards and samples must be diluted with the Sample Diluent containing 2-vinylpyridine.

### 4.10. Immunofluorescence Assay

AGS cells (7 × 10^4^) were seeded on 20 mm coverslips. After 24 h, the cells were challenged (for 3 h) with the compounds: CDDP (20 µM), P1 (20 µM), and P2 (10 µM). The cells were then fixed in 4% formaldehyde for 15 min, washed with PBS, permeabilized with 0.2% Triton for 5 min, and finally blocked with 1% BSA for 1 h. DNA damage was evaluated by detecting the foci per nuclei in coverslips incubated for 1 h with the primary antibody H2AX Ser139 (Cell Signaling: #2595; Cell Signaling Technology, Inc., Danvers, MA, USA) at room temperature, followed by a 1 h incubation with the appropriate secondary antibody. DNA was stained with DAPI to visualize nuclei. Fluorescence microscopy was performed using a NIKON Eclipse 90i (Nikon Instruments Inc., Melville, NY, USA). The image analysis was performed using the software program Nikon NIS-Elements and Image J.

### 4.11. Mitochondrial Membrane Potential

AGS cells were seeded at a density of 3 × 10^5^ cells in 15 µm 8-well IBIDI plates and incubated overnight. The cells were then treated with compounds at their IC_50_ concentrations and incubated for 2 h. Subsequently, probes were incubated with cells for 30 min at 37 °C at a concentration of 100 nM for Mitotracker Green (MTGreen, M7514, Invitrogen ThermoFisher, Waltham, MA, USA) and 20 nM Mitotracker Red CMXRos (CMXROS, M7512, Invitrogen). Fluorescence was detected using a confocal microscope LSM710, Zeiss, objective plan-apochromatic 63×). The image analysis was performed using the software program ImageJ. To determine mitochondria functionality, the ratio between ΔΨ_m_ (MitoTracker Red CMXRos) versus mitochondrial mass (MitoTracker Green) was calculated.

### 4.12. Western Blotting

AGS cells were seeded at a density of 8 × 10^5^ cells in p60 plates and incubated overnight. The cells were then treated with compounds (CDDP 20 µM, P1 20 µM, and P2 10 µM) for 3, 6, and 24 h. Total protein extracts were obtained using the previously described lysis buffer—25 mM HEPES pH 7.5, 0.3 M NaCl, 1.5 mM MgCl_2_, 0.2 mM EDTA, 0.5 mM DTT, 20 mM β-glycerophosphate, 0.1 mM Na_3_VO_4_, 0.1% triton X-100 [54]—in the presence of protease inhibitors (PPC1010-5ML, Sigma-Aldrich). Twenty micrograms of protein per sample was loaded and resolved using 10%, 12%, or 15% SDS-PAGE polyacrylamide gels and then transferred onto PVDF membranes, followed by immunodetection using appropriate antibodies, purchased from Santa Cruz Technology, Santa Cruz, CA, USA: Mcl-1 (sc-819), p62/SQSTM1 (sc-28359), Nrf2 (sc-518033, 1:500); Cell Signaling Technology, Inc. Danvers, MA Boston, USA: BIM (#2933), BAX (#5023), BAK (#12105), p-p53^ser15^ (#9284), p-eIF2α (#9721), GAPDH (#2118, 1:5000); Bio-Techne, Minneapolis, MN, USA: CHOP (#BP2-13172, 1:2500), GRP78 (#BP1-06277), XBP1 (#BP1-77681, 1:5000), ATF6 (#BP1-40256); or Sigma-Aldrich: α-tubulin (1:10,000, #T9026), LC3 (#L8918). Unless indicated, all antibodies were diluted 1:1000. Secondary antibodies conjugated with horseradish peroxidase were purchased from BioRad, Madrid, Spain, and chemiluminescence detection was performed using ECL (Santa Cruz Biotechnology).

### 4.13. RT-qPCR

AGS cells were seeded at a density of 8 × 10^5^ cells in p60 plates and incubated overnight. The cells were then treated with compounds (CDDP 20 µM, P1 20 µM, and P2 10 µM) for 24 h. Total cellular RNA was extracted using Tri-Reagent (Life Technologies, ThermoFisher, Waltham, MA, USA), following the manufacturer’s instructions. One microgram of total RNA was primed with random primers and cDNA synthesized with an M-MLV reverse transcriptase following the manufacturer’s procedure (Promega). Target genes were amplified using a SYBR Green polymerase chain reaction assay, working with the specific primer sets listed in Table 3:

Thermal cycling of the qPCR reaction was initiated with a denaturation step at 95 °C for 10 min. The process consisted of 40 cycles (denaturation at 95 °C for 15 s, annealing at 60 °C for 30 s, and elongation at 75 °C for 30 s). PCR amplifications were carried out in a StepOne Real-time PCR System (Applied Biosystems, ThermoFisher, Waltham, MA, USA, 4376357). Relative mRNA levels were calculated using the delta-Ct method (2^−ΔΔCt^), where each 1-Ct difference equals a two-fold change in transcript abundance, using GAPDH as an endogenous reference. ΔΔCT represents the difference between the mean ΔCT value of the cells tested and the mean ΔCT value of the calibrator, both calculated for the same PCR run.

### 4.14. Statistics and Reproducibility

Statistical analyses were performed using GraphPad prism 8.0 Student’s 2-tailed *t*-test and one-way ANOVA with a Dunnett post-test. Values of * *p* < 0.05 were considered significant.

## Figures and Tables

**Table 1:** IC_50_ concentration (mean value ± SD) µM at 48 h. N = 3. Selective indexes in reference to the non-tumor gastrointestinal cells (HPDE) are included in brackets.

	AGS	MKN45	PANC1	AC16	HPDE
CDDP	24.95 ± 2.78 (0.1)	7.22 ± 0.72 (0.2)	9.71 ± 1.21 (0.2)	6.51 ± 1.87	1.58 ± 0.89
P1	9.12 ± 0.59 (>2.7)	20.91 ± 1.73 (>1.2)	10.57 ± 2.43 (>2.4)	19.91 ± 2.89	>25
P2	5.66 ± 0.41 (3.9)	10.15 ± 0.13 (2.2)	9.74 ± 0.50 (2.3)	7.57 ± 1.64	22.09 ± 0.53

**Table 2:** Subcellular distribution of platinum in AGS cells after 3 h of 10 µM treatment with P1 and P2. The data are expressed as the mean values (±SD) of the metal (ng/mL) content in the nucleus or cytoplasm. N = 3.

	Pt (ng·mL^−1^)	
	Nucleus	Cytoplasm
Control	<0.05	<0.05
P1	0.54 ± 0.24	0.87 ± 0.18
P2	1.35 ± 0.07	2.25 ± 0.64

**Table 3:** Primer sequences for the RT-qPCR analysis.

Primer	Sequence
*HSPA5*	F: TGCCCAACGCCAAGCAACC R: AATAGCAGCTGCCGTAGGCTCG
*ERN1*	F: ATCTTGGGCGAACAGAATACACC R: CACCGGAGCTCTCGGGTTTTG
*XBP1*	F: GCTTCTGTCGGGGCAGC R: ACTCTGTTTTTCAGTTTCCTCCTCA
*EIF2AK3*	F: GGGAGCAGGGAAGAAAAGGTCA R: ACACCAAGGAACCGGATCCCAC
*ATF4*	F: GGGCTCCTCCGAATGGCTGG R: CGGAGAAGGCATCCTCCTTGC
*DDIT3*	F: ACCTCCTGGAAATGAAGAGGAAGAA R: GGGCTCTGGGAGGTGCTTGT
*ATF6*	F: AACCTGCACCCACTAAAGGCCA R: TCCCCCAGCAACAGCAAGGAC
*GAPDH*	F: GAGAGACCCTCACTGCTG R: GATGGTACATGACAAGGTGG

## Data Availability

All data generated or analyzed during this study are included in this published article and its Supplementary Information files. All the uncropped blots are collected in Figure S6.

## Supplementary Materials

The following supporting information can be downloaded at https://www.mdpi.com/article/10.3390/ijms25147739/s1.
