# Supplementary material for: Trans-[Pt(amine)Cl2(PPh3)] Complexes Target Mitochondria and Endoplasmic Reticulum in Gastric Cancer Cells
Source: Int J Mol Sci. 2024 Jul 15;25(14):7739. doi: 10.3390/ijms25147739 (PMC11276749; doi:10.3390/ijms25147739)
Supplement: Supplementary file 1 [file ijms-25-07739-s001.zip › ijms-3045316-supplementary.pdf]

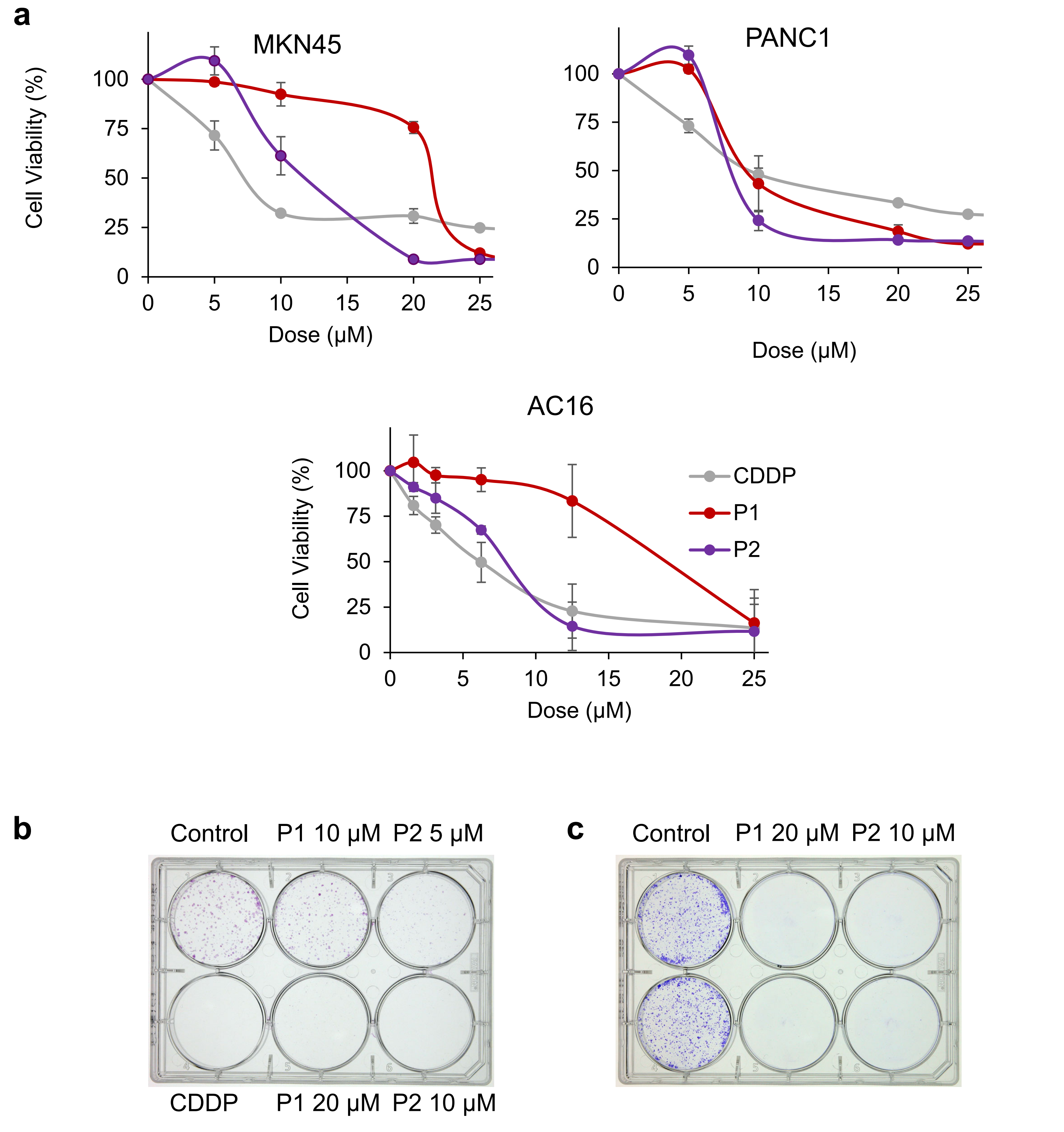

Figure S1. Phosphine agents decrease cell proliferation. **a** Cell viability studies with P1 (red), P2 (purple) and CDDP (grey) used as a reference, in MKN45, PANC1 and in healthy cells AC16 after 48h-treatment. Cells were treated with increasing concentrations (0−25 µM). The percentage of viable cells was quantified by MTS assay. Data represent the mean values obtained in three experiments performed in quadruplicate. **b** Representative images of Colony Forming Unit (CFU) assay used to determine cell clonogenicity (quantification depicted in Figure 1b). AGS cells were treated with CDDP (20 µM), P1 (10 and 20 µM) or P2 (5 and 10 µM), and colonies formed were stained with crystal violet and quantified 10 days later. **c** Representative images of Colony Forming Unit (CFU) assay with a 3h-pre-treatment (Quantification depicted in Figure 1c) with P1 (20 µM) and P2 (10 µM).

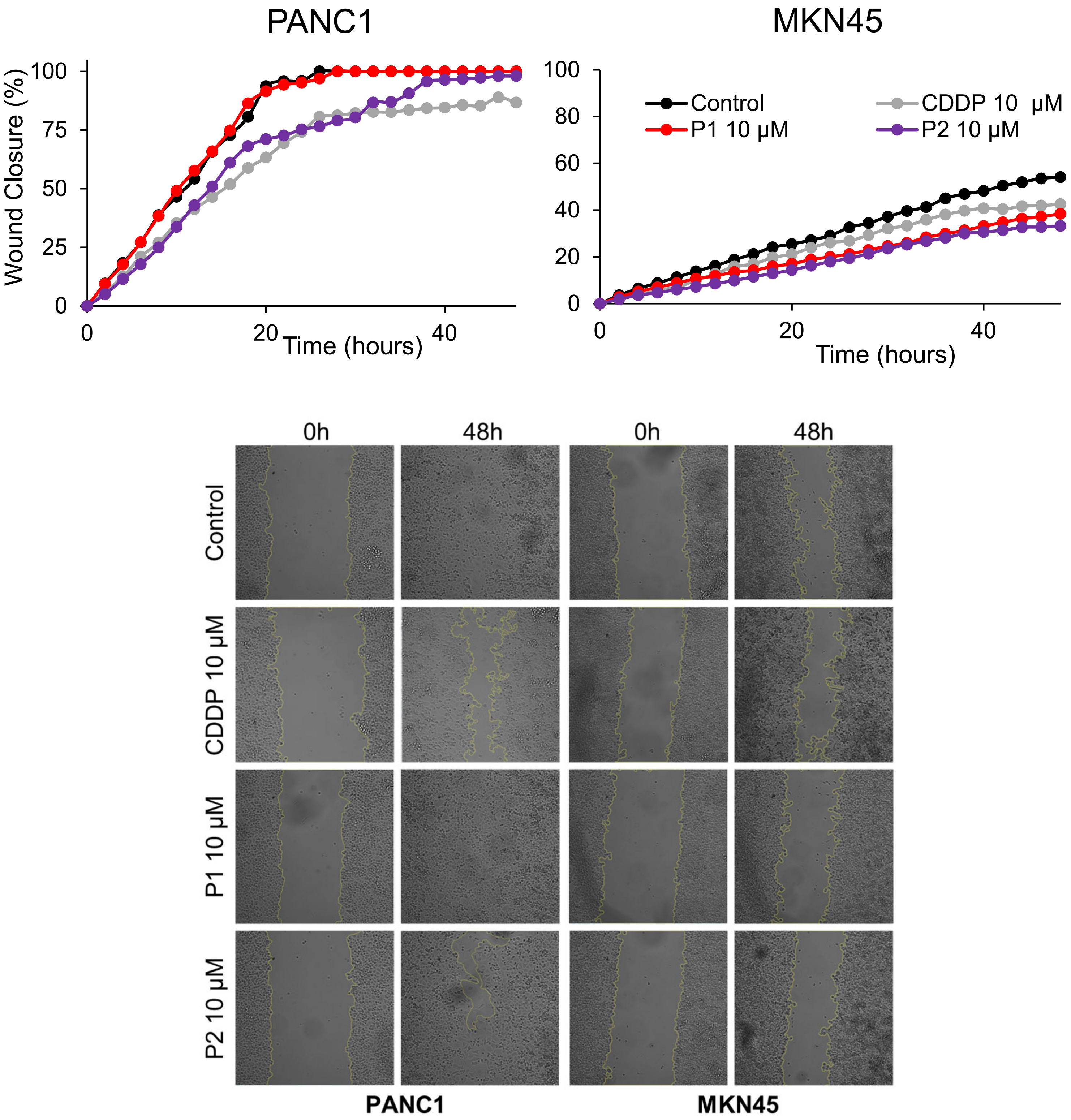

Figure S2. P1 and P2 do not interfere in cell migration. PANC1 and MKN45 cells were treated with CDDP, P1 and P2 (10 μM). The graph shows the percentage of wound closure over the study time using the ImageJ program. Control (black), CDDP (grey), P1 (red) and P2 (purple). Lower panel: Representative images of the first (0 h) and last picture (48 h) of each condition taken during the wound healing experiment. Images were taken at 10× magnification, every 2 h for 48 h. The yellow line represents the wound border**.**

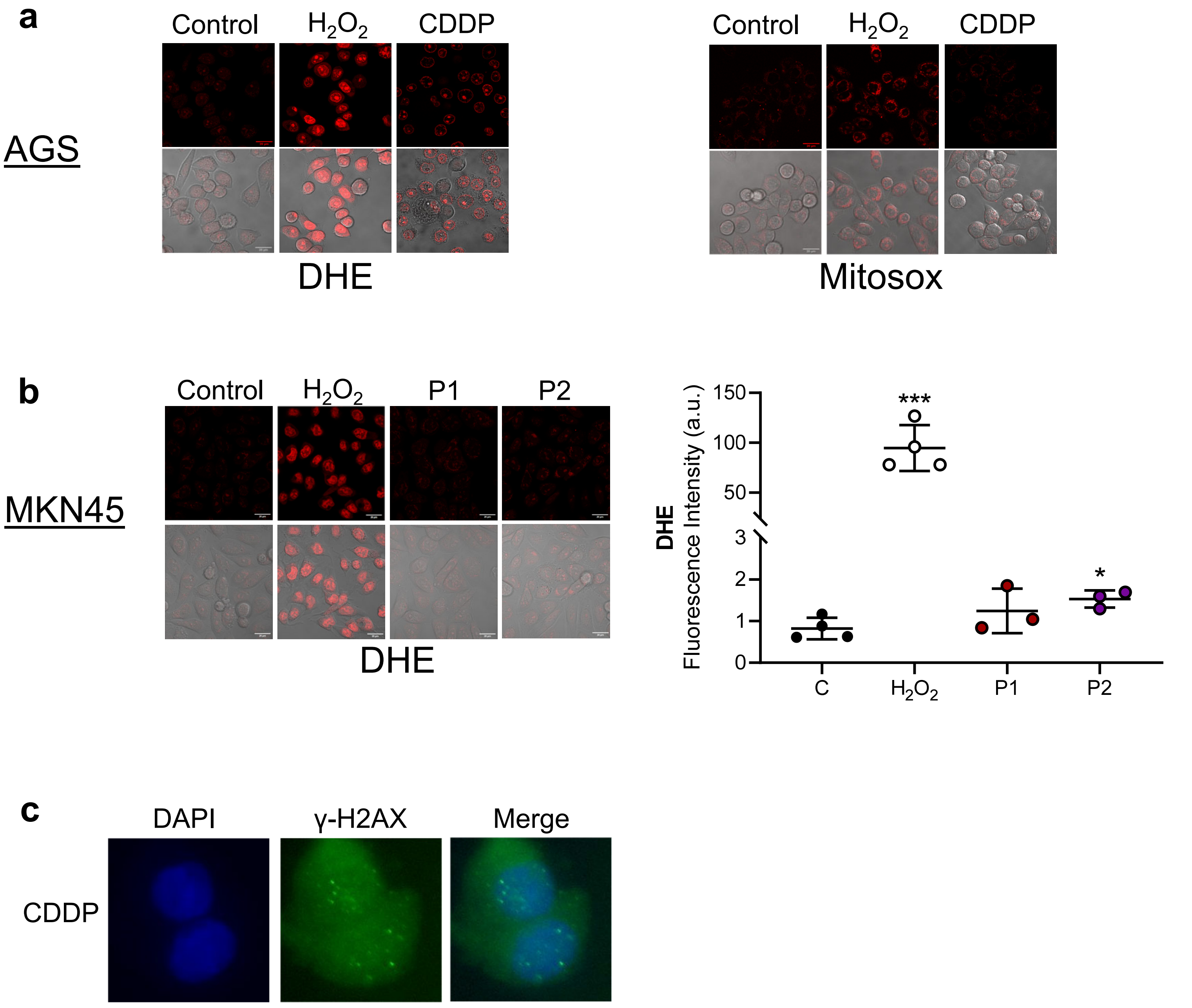

Figure S3. a Representative images of the quantification by confocal microscopy of ROS generation after 1 h-treatment with H_2O_2 (200 µM) and CDDP (10 µM) using DHE and MitoSox as $O_2^{\bullet -}$ fluorescence indicator, in cytosol and mitochondria, respectively. Scale bar represents 20 µm. **b** Detection of ROS in MKN45 cells after the treatment with H_2O_2 (positive control, 200 µM), P1 (20 µM) and P2 (10 µM) by confocal microscopy using DHE. Cells were treated with the compounds for 1 h followed by 30 min of incubation with the probe. Representative images of each condition were taken. Scale bar represents 20 µm. Fluorescence Intensity (per cell) was quantified and depicted in the graph. Statistical significance was by evaluated one-way ANOVA with Dunnett post-test (*p<0.01, ***p<0.001) compared to the untreated cells (C: Control). N=3. **c** Representative images of AGS cells treated with CDDP (20 µM).vγ-H2AX foci (green fluorescence) were detected by immunofluorescence using DAPI to stain nuclear DNA (blue fluorescence). Scale bar: 20 µm.

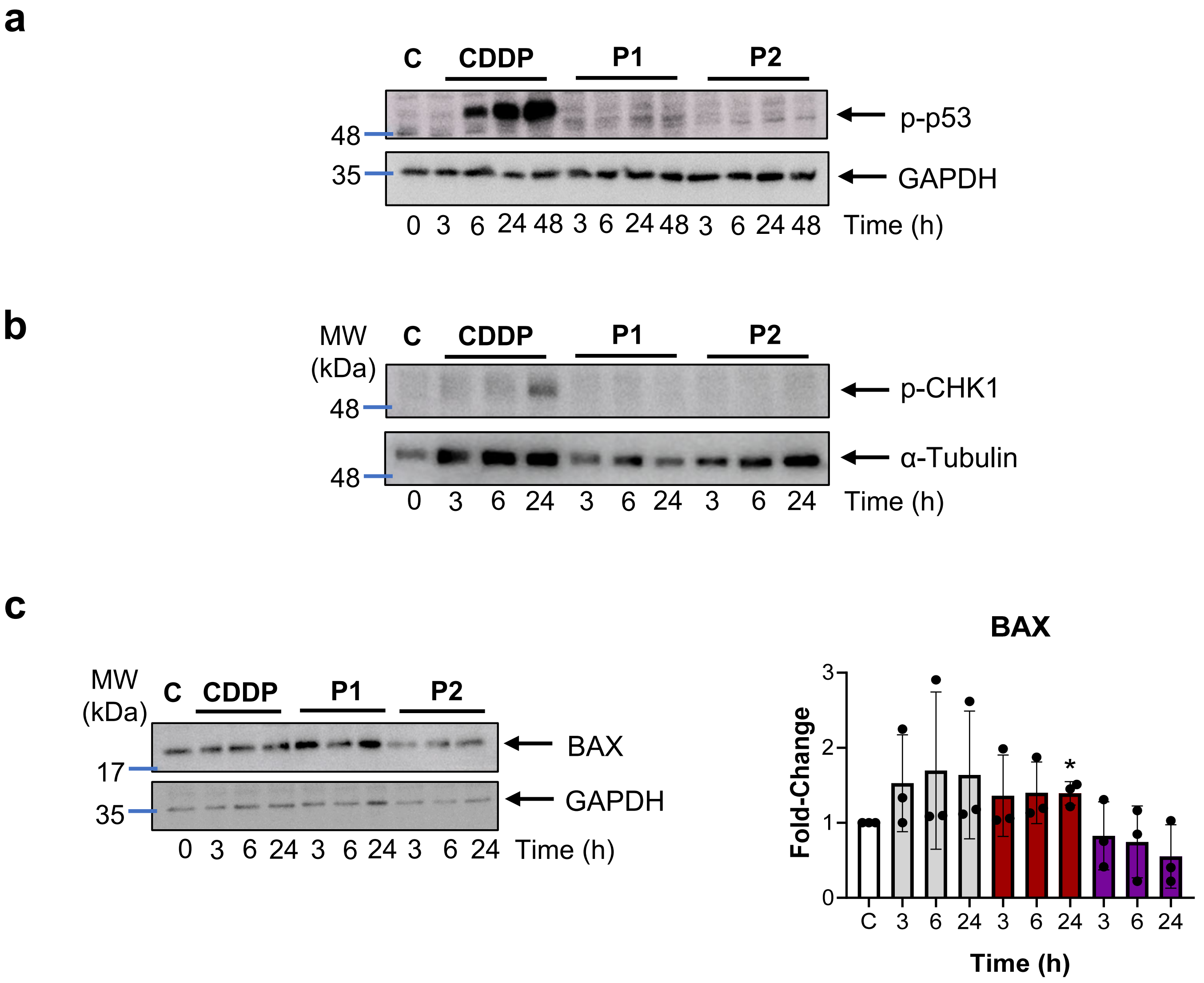

Figure S4. Western Blot Analysis. a Phosphorylation of p53. **b** Phosphorylation of CHK1. **c** Intrinsic apoptosis measuring BAX. Representative western blots in AGS cells after the treatment with IC_{50} concentration of CDDP, P1 or P2 at different times (3, 6 and 24 h). GAPDH was used as an endogenous loading control. From BAX graph show the mean ± SD densitometric analyses of each protein normalized with GAPDH from three independent experiments by using ImageJ (Area under the peak method), Control cells (C, white bars), CDDP (grey), P1 (red) and P2 (purple). The statistical significance was evaluated with Student's 2-tailed t-test (*p<0.05) compared to the untreated cells (C: Control) set as 1.0.

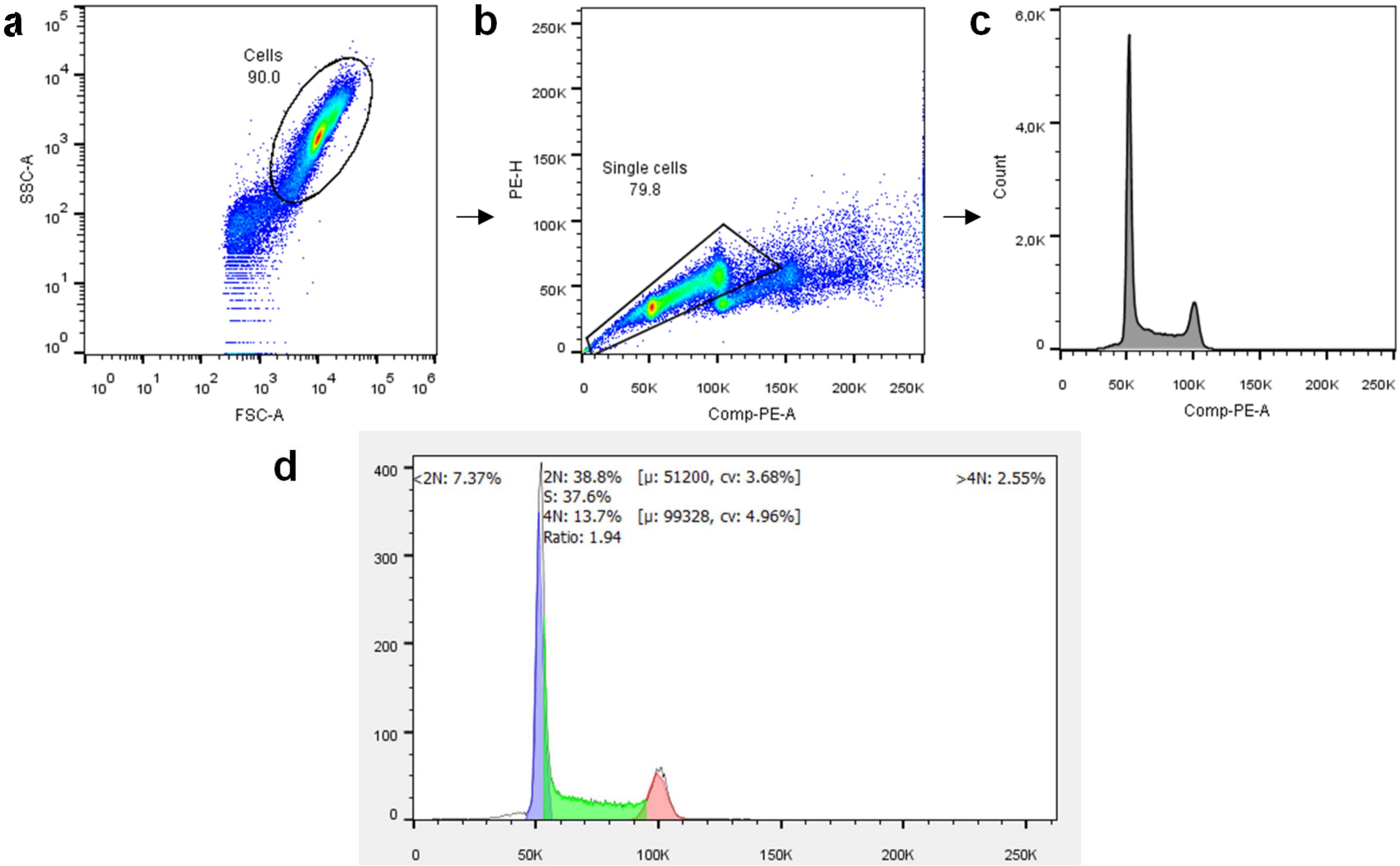

Figure S5. Flow cytometry analysis. **a** Cell region selection by creating a density dot plot facing SSC and FSC. **b** With the cells selected, a density dot plot (PE-H vs. PE-A) is created to determine the single cell region needed to represent the histogram in figure **c**. **d** Statistical analysis created with the previous histogram adjusting the 2n (in blue), S (in green) and 4n (in pink) regions for correct processing.

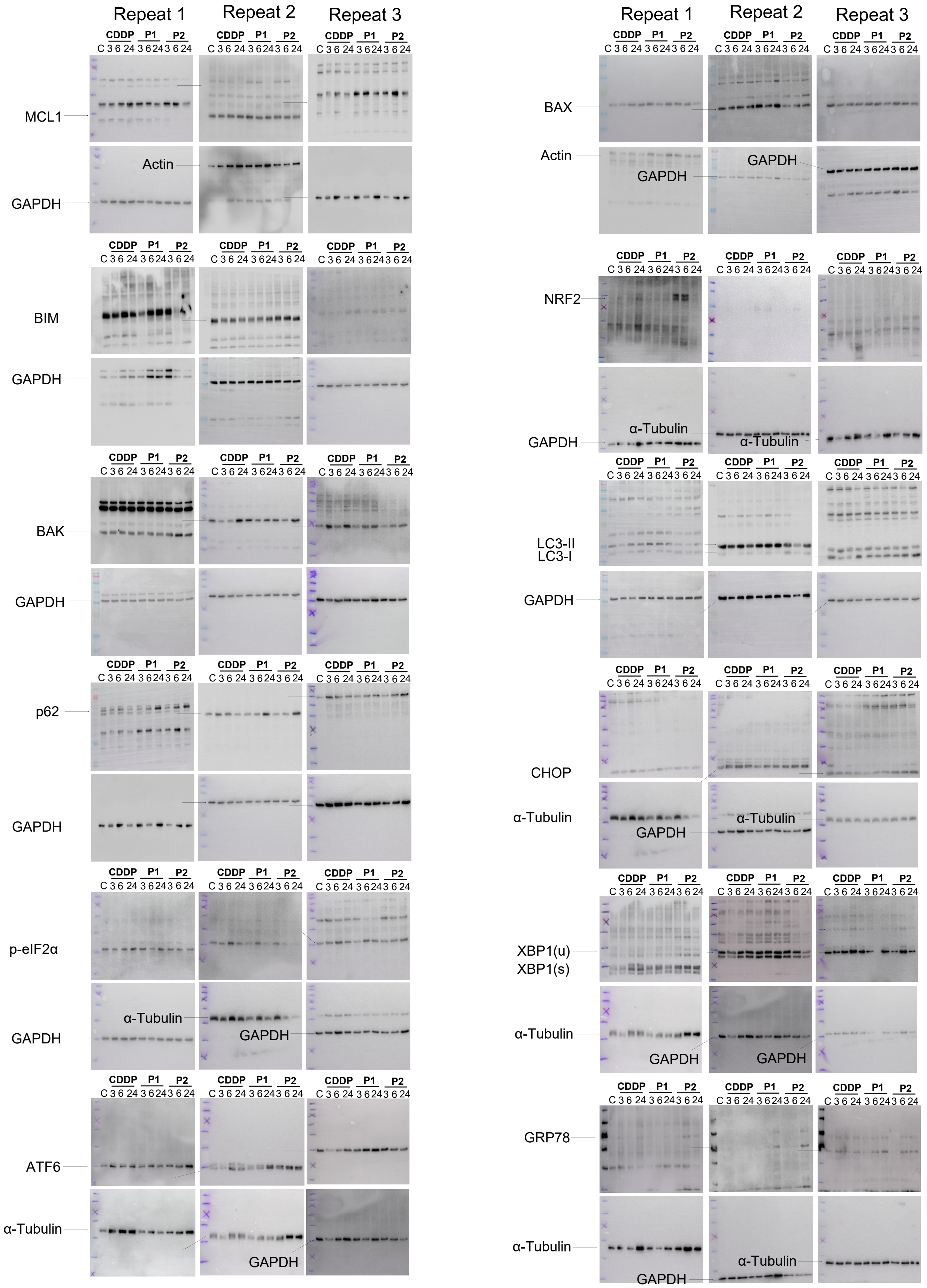

Figure S6. Uncropped Western Blot images.

Table S1. Selective indexes with respect to the non-tumor AC16 cells .N=3.

	**AGS**	**MKN45**	**PANC1**
**CDDP**	0.3	0.9	0.7
**P1**	2.2	1.0	1.9
**P2**	1.3	0.7	0.8

Table S2. Percentage of cells (mean ± SD) in each Cell Cycle Phase after 24 h-treatments. N=3.

	**< 2N**	**G0/G1**	**S**	**G2/M**	**> 4N**
**Control**	4.3 ± 1.7	43.6 ± 6.9	28.5 ± 3.9	21.4 ± 7.0	2.2 ± 0.4
**P1 10 μM**	6.8 ± 3.1	42.4 ± 8.0	34.1 ± 7.2	14.0 ± 2.1	2.8 ± 1.9
**P1 20 μM**	8.4 ± 5.2	45.1 ± 8.0	29.8 ± 5.8	15.6 ± 5.5	3.1 ± 2.2
**P1 30 μM**	7.2 ± 3.4	47.5 ± 9.4	28.8 ± 8.0	19.0 ± 8.2	4.0 ± 3.2
**P2 10 μM**	7.9 ± 3.7	38.6 ± 7.9	34.3 ± 5.3	15.8 ± 4.5	3.2 ± 2.2
**P2 20 μM**	9.2 ± 3.0	29.7 ± 10.0	27.9 ± 7.6	24.7 ± 7.9	3.2 ± 1.4
**P2 30 μM**	10.9 ± 4.7	25.9 ± 3.6	33.0 ± 7.9	28.9 ± 7.5	3.5 ± 0.6
